# Real-Time Terrain Storage Generation from Multiple Sensors towards Mobile Robot Operation Interface

**DOI:** 10.1155/2014/769149

**Published:** 2014-07-02

**Authors:** Wei Song, Seoungjae Cho, Yulong Xi, Kyungeun Cho, Kyhyun Um

**Affiliations:** ^1^College of Information Engineering, North China University of Technology, Beijing 100144, China; ^2^Department of Multimedia Engineering, Dongguk University, Seoul 100-715, Republic of Korea

## Abstract

A mobile robot mounted with multiple sensors is used to rapidly collect 3D point clouds and video images so as to allow accurate terrain modeling. In this study, we develop a real-time terrain storage generation and representation system including a nonground point database (PDB), ground mesh database (MDB), and texture database (TDB). A voxel-based flag map is proposed for incrementally registering large-scale point clouds in a terrain model in real time. We quantize the 3D point clouds into 3D grids of the flag map as a comparative table in order to remove the redundant points. We integrate the large-scale 3D point clouds into a nonground PDB and a node-based terrain mesh using the CPU. Subsequently, we program a graphics processing unit (GPU) to generate the TDB by mapping the triangles in the terrain mesh onto the captured video images. Finally, we produce a nonground voxel map and a ground textured mesh as a terrain reconstruction result. Our proposed methods were tested in an outdoor environment. Our results show that the proposed system was able to rapidly generate terrain storage and provide high resolution terrain representation for mobile mapping services and a graphical user interface between remote operators and mobile robots.

## 1. Introduction

In recent times, technologies for dynamic terrain reconstruction and modeling using multiple sensors have been extensively researched in order to provide mobile vehicles with the ability to conduct free-space detection and support collision-free navigation [1]. In such applications, datasets received from multiple sensors, including 3D point clouds, video images, global positioning system (GPS) data, and rotation states, are integrated to produce accurate and reliable terrain information.

Light detection and ranging (LiDAR) sensors are widely used to measure distances and capture 3D surfaces using lasers, such as Velodyne [2] and Sick. These sensors collect 3D point clouds that do not contain any color or texture information owing to the characteristics of the laser. As a result, synchronized video images are required to map the color information to the sensed 3D point cloud, thus realizing intuitive terrain visualization towards mobile mapping services.

Conventional real-time visualization systems mostly apply a voxel map or a color mesh to represent a terrain model. A voxel map is generated by integrating the sensed 3D point clouds into regular grids. From the voxel map, a terrain mesh is generated by integrating the top points in the* x*-*z* cells into a regular triangular mesh [3]. These methods allocate one color per voxel or vertex so that the resolution of the terrain model is low.

In order to improve terrain visualization so as to rapidly obtain an intuitive representation, real-time terrain modeling and photorealistic visualization systems have been developed [4]. A photorealistic visualization attempts to realistically represent a 3D terrain and object models in the virtual world [5]. To improve the visualization speed, real-time terrain visualization methods have been studied [6, 7]. In recent times, with the rapid development of graphics cards, a graphics processing unit (GPU) with a highly parallel many-core architecture has been widely used in 2D image processing, 3D data analysis, visualization, and other fields [8, 9]. GPU programming can be used to realize a high-speed and high-quality large-scale terrain reconstruction system [10].

Our study aims to reconstruct an intuitive terrain model from large-scale datasets using limited memory for providing mobile robot operator with a graphical user interface (GUI) of surrounding environment. Typically, the captured video images and sensed point clouds are registered into the terrain model. However, when the sensed datasets are registered incrementally, they become very large in size and exceed the computer memory capacity. Furthermore, the large computational cost incurred for processing large-scale datasets makes terrain modeling and visualization slow. Therefore, it is necessary to develop an effective redundancy removal method for reducing the size of the 3D point cloud in order to realize real-time large-scale terrain reconstruction.

In this paper, we describe a real-time terrain storage generation and intuitive representation system using multiple sensors. The first step for real-time terrain modeling is a redundancy removal method for the large-scale 3D point cloud. To register the sensed datasets into the terrain model having limited memory, we develop a voxel-based flag map as a comparative table for removing redundant points. The compressed point clouds are registered into a nonground point database (PDB) and ground texture database (MDB) using a height histogram method [11]. To visualize the reconstructed terrain model, the nonground PDB is represented using a voxel map, generated by integrating the nonground 3D points into regular grids. The ground MDB is implemented as a node-based terrain mesh. Each node in the mesh contains a certain number of ground surface vertices and a node texture. The node textures are integrated to form a texture database (TDB). To realize real-time TDB generation, the GPU is used to map the triangles of the node texture in parallel. Finally, we represent the reconstructed terrain model by rendering the points in the nonground PDB and overlaying the MDB with the TDB.

The proposed real-time terrain storage generation technique allows a mobile robot to survey, navigate through, and interact with its environment by providing quickly accessible and accurate information regarding the surrounding terrain [12].

The remainder of this paper is organized as follows. In Section 2, we survey related works on terrain modeling and representation methods. In Section 3, we explain the terrain storage generation and representation system. In Section 4, we analyze and evaluate the performance of the proposed multithread-based terrain storage generation system. Finally, in Section 5, we present our conclusions.

## 2. Related Work

During navigation and interactive tasks, rapid feedback on intuitive representations of a robot's surrounding terrain is required for real-time operation. Conventionally, a voxel map and textured mesh have been applied for this type of terrain modeling. Rovira-Más et al. [13] applied a voxel map to represent a reconstructed terrain model. However, they allocated only one color per voxel, which caused distortions. Sukumar et al. [14] integrated sensed datasets into a texture mesh for terrain reconstruction. However, it is difficult for these systems to process large-scale datasets of the kind obtained in outdoor environments in real time.

To realize real-time transmission and registration of large-scale point clouds with limited memory, researchers investigated data redundancy removal methods to reduce the buffer size of the terrain model [15, 16]. Kammerl et al. [17] applied a voxel map to quantize points into regular grids in order to compress 3D point clouds. The generated voxel map is stored on a hard disk by using an octree data structure. Although this method employs lossy compression, it efficiently removes redundant data using a traversing process to compare whether or not this point is stored. In a large-scale environment with a low-density point cloud, the depth of an octree is large, which causes high data searching complexity. Thus, the data size of each node in an octree is large, which leads to a low compression ratio. For a large-scale terrain model, this traversing process requires substantial computational power. Therefore, an effective and rapid data compression method is necessary for real-time data transmission.

Gingras et al. [18] reconstructed an unstructured surface from a 360° point cloud scan and represented the traversable areas using a compressed irregular triangular mesh. They applied a mesh simplification algorithm to reduce the number of triangles on the large-scale terrain surface. Zhuang et al. [19] proposed an edge-feature-based iterative closest point (ICP) algorithm to extract edge points, which were registered on a 3D roadmap integrated with planar features and elevation information. By using this method, a large number of redundant points of pseudoedges could be removed to realize rapid large-scale point cloud registration. However, the visualization achieved in these studies was not sufficiently intuitive for unmanned vehicle operations. To overcome this limitation, it is necessary to overlay the 3D terrain model with captured video images.

Meanwhile, the image buffers of the reconstructed terrain model become increasingly full when a mobile robot navigates in a large-scale environment. Even when using compression algorithms, such as Huffman coding, JPEG, wavelet filter, and MPEG for 2D images [20, 21], the buffers of the captured video images are registered incrementally and they ultimately become so large that they exceed the memory capacity of the mobile robot. An effective and rapid image registration method is therefore necessary to realize intuitive terrain reconstruction with limited memory consumption.

In this study, we use a voxel map and texture mesh to represent nonground and ground terrain information separately. To improve the performance of terrain storage on the hard disk, we propose a terrain storage generation and updating method that does not need traverse elements stored on the hard disk, thus realizing improved speed. To compress video buffers, we present a TDB generation method by integrating several captured images into a node texture.

## 3. Terrain Storage Generation from Multiple Sensors

In this section, we develop a terrain storage generation system in which we register large-scale datasets into a terrain model with limited memory in real time. In this system, we employ a 3D point cloud compression method that uses a voxel-based flag map. Then, we register nonground points into a voxel map and ground points into a texture mesh. Next, we employ a GPU-based mapping algorithm to convert the 3D mesh triangle into a 2D image. Finally, the intuitive visualization process is implemented by rendering the generated terrain storage.

### 3.1. Terrain Storage Generation and Representation System

**Figure 1:**
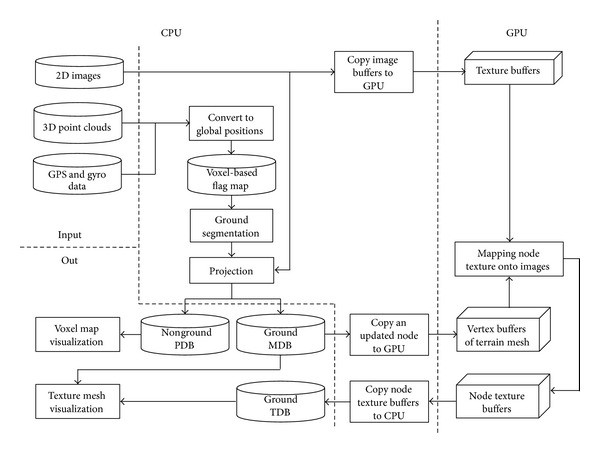
Framework of terrain storage generation system.

In this section, we describe the multithread-based terrain storage generation system for mobile robots, as shown in Figure 1. This system involves several processes such as data collection, dataset compression, nonground PDB generation, ground MDB, and TDB generation.

A mobile robot collects 3D point clouds, 2D images, GPS, and rotation states as a real-world interface. The received 3D points are converted into global positions on the basis of the GPS and rotation states. By quantizing the 3D point clouds into regular voxels, we create a voxel-based flag map to remove redundancies.

Some nonground objects have overhanging parts such as roofs and leaves. It is difficult to represent these objects using a height map. In this study, we propose the use of a voxel map to represent nonground objects and a terrain mesh to represent the ground surface. Therefore, we classify these points into ground and nonground using the height histogram method [11] before terrain modeling.

The color data of the nonground voxels in the PDB are computed by the projection from the 3D voxels to the sensed 2D image. From the nonground points, we select a vertex as the top point in an* x-z* cell and insert it into a terrain mesh. To realize intuitive visualization, we create a texture triangle of several pixels for each triangle in the MDB by mapping the captured images onto the mesh. We apply GPU programming to map these texture triangles that are combined into a TDB. By mapping the generated TDB onto the mesh, a textured mesh is represented.

### 3.2. Voxel-Based Flag Map

We developed a voxel-based flag map to register 3D points into the terrain model without reduplication. To realize real-time terrain modeling, we register a point into the flag map based on the spatial relation without a traversal process.

The coordinate system of a sensed 3D point *p*(*x*, *y*, *z*) is centered at the robot position *L*. Before inserting this point into the flag map, we convert it to a relative position based on the coordinate system at the center of the flag map. The converted coordinate is formulated as follows:

(1) $$\begin{array}{c} p^{\prime }=R\left(p+L_{c}\right)+L-L_{0}, \end{array}$$

where *L*_*c*_  is the 3D vector from the 3D sensor location to the GPS sensor location, *L*_0_ is the center of the flag map, and *R* is the mobile rotation matrix.

**Figure 2:**
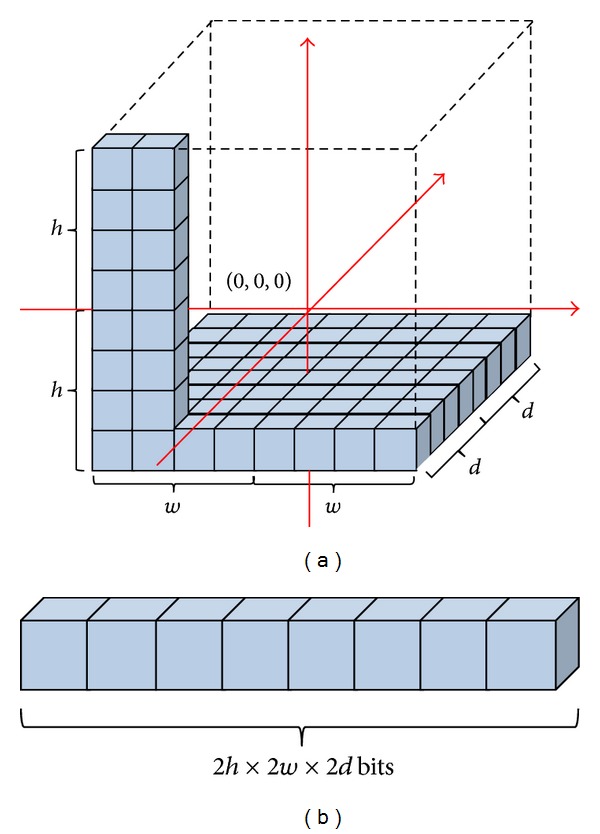
Definition of voxel-based flag map. (a) A space of regular voxels. (b) A buffer stream of 2*h* × 2*w* × 2*d* bits.

Subsequently, the converted positions are quantized into a space of regular voxels, as shown in Figure 2(a). The constants *w*,  *h*, and *d* are the maximum measurements along the *x*-, *y*-, and *z*-axes of the flag map, respectively. The size of the voxel is defined as *μ*. In this manner, a flag map has 8*hwd* grids, which represents a space of 8*hwd* *μ* ^3^ m^3^. Subsequently, we specify a bit-stream to define a voxel-based flag map, as shown in Figure 2(b). If the coordinates of *p*′(*x*′, *y*′, *z*′) satisfy |*x*′| < *wμ*, |*y*′| < *hμ*, |*z*′| < *dμ*, the voxel index mapped from *p*′ is formulated as follows:

(2) $$\begin{array}{c} v=4wh\left\lfloor \frac{z^{\prime }}{\mu }+d\right\rfloor +2h\left\lfloor \frac{x^{\prime }}{\mu }+w\right\rfloor +\left\lfloor \frac{y^{\prime }}{\mu }+h\right\rfloor , \end{array}$$

where the function ⌊*N*⌋ returns the largest integer that is less than or equal to *N*.

We allocate a 1-bit variable *f*(*v*) for each voxel *v*. We initialize the flag map by specifying *f*(*v* ∈ *V*) = 0, where the set *V* = {*v* ∈ [0,2*h* × 2*w* × 2*d*)}. When at least one point exists in the covered area of the voxel *v*, *f*(*v*) = 1; otherwise, *f*(*v*) = 0. After the robot collects several consecutive frames of 3D point clouds, some points are inserted into the same voxel. This causes wasteful duplication of memory if we register these points into the terrain model. To remove redundant points, we register voxels when they are sensed for the first time. Hence, when a new point is converted to a voxel *v* and *f*(*v*) = 1, the robot does not register this point in the terrain model.

We register voxels when they are sensed for the first time in order to avoid redundancy. After generating a flag map from several consecutive frames of 3D point clouds, we find that covered voxels exist between the current and the previous sensed frames.

For long-term navigation in an environment, the range of the sensed points exceeds the defined range of the flag map. To solve this problem, we shift the center of the flag map to the position of the vehicle when the vehicle moves to a certain distance. It is necessary to drop the passing information from the memory of the flag map and store new sensed points. In this manner, we utilize a flag map with limited range to represent the information about the dynamic environment surrounding the robot.

### 3.3. Nonground and Ground Terrain Modeling

**Figure 3:**
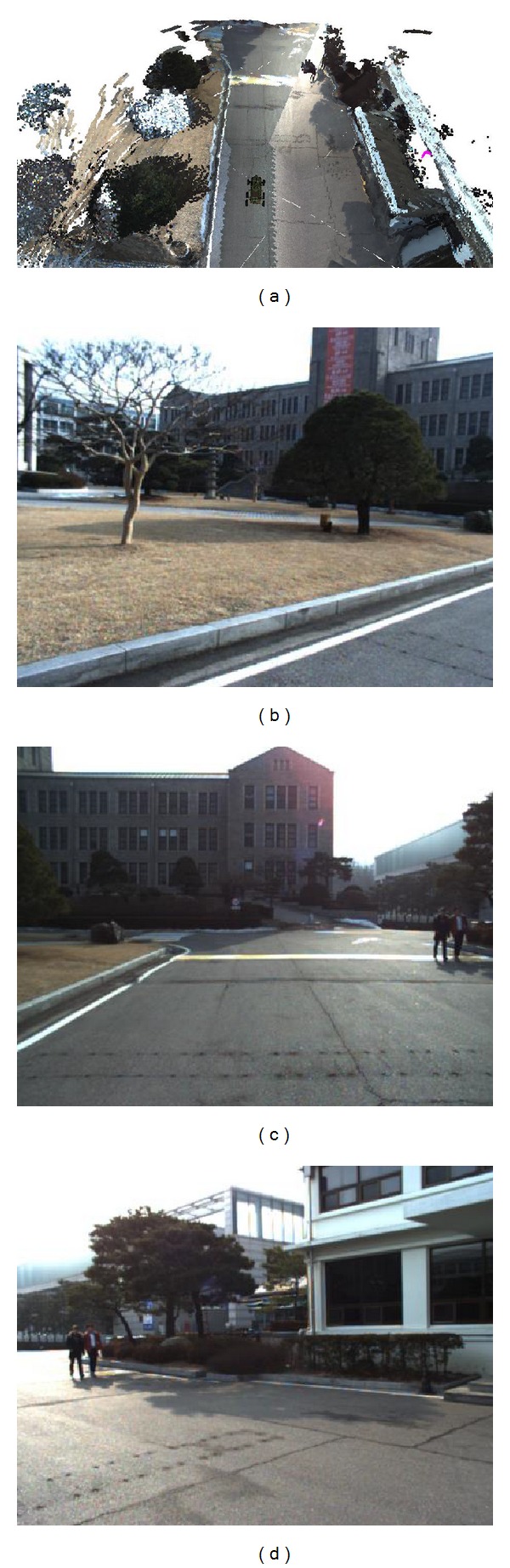
A terrain reconstruction model. (a) Nonground objects and ground surface representation. (b)–(d) Captured images.

It is impossible to sense the points below the ground surface using 3D sensors such as LiDAR sensors. The sensors provide the top points of the ground surface and the surface points of nonground objects. To represent an intuitive ground surface, we apply a texture mesh by mapping the texture onto a digital elevation model (DEM). The road and grass group in Figure 3(a) are represented using a texture mesh. This method provides large-scale terrain with low memory consumption. However, it is difficult to represent objects that have overhanging parts, such as roofs and leaves. To realize real-time terrain modeling, we apply a color voxel map consisting of a list of voxels to represent nonground objects. The trees and building in Figure 3(a) are represented using a color voxel map.

Before terrain modeling, it is necessary to segment the registered points into ground surface and nonground objects using the voxel-based flag map method. Based on the spatial distribution of ground surface and nonground objects, we segment the ground surface using the height histogram method described in [11], which is a fast and dynamic ground segmentation method. The classified nonground and ground voxels are registered into the PDB and the MDB, respectively.

Owing to the characteristics of a LiDAR sensor, the sensed points contain no color information. This makes remote operation inconvenient and less perceptual. To represent the terrain model world with its real appearance, we project the 3D voxels of PDB and the vertices of MDB to the captured 2D images, as shown in Figures 3(b)–3(d).

### 3.4. TDB Generation

**Figure 4:**
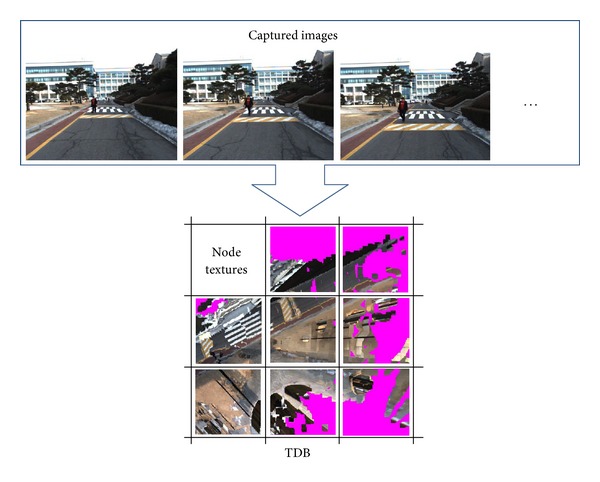
TDB generation from captured images.

A terrain mesh is always generated by integrating the top points in the* x-z* cells into a regular triangular mesh. By overlaying the 3D terrain mesh with captured video images, the visualization system provides perceptual imagery of the 3D terrain geometrical model, as shown in Figure 4.

We represent the nonground MDB using a node-based texture mesh. The mesh is generated using several nodes. In our application, each node has 128 × 128 textured vertices, and the cell size is 0.1 × 0.1 m^2^. The height value of each vertex in the mesh is updated with the registered ground 3D voxels. If a new 3D voxel is to be inserted into the reconstructed terrain mesh but is outside the existing nodes, we create a new node to register this voxel.

To represent an intuitive ground surface, we traditionally map the captured images onto the terrain mesh. However, the sensed images are registered incrementally, which become so large that they exceed the memory capacity. To reconstruct a texture terrain mesh using limited memory, we propose a TDB generation method, registering several captured images into node textures without redundant pixel buffers.

**Figure 5:**
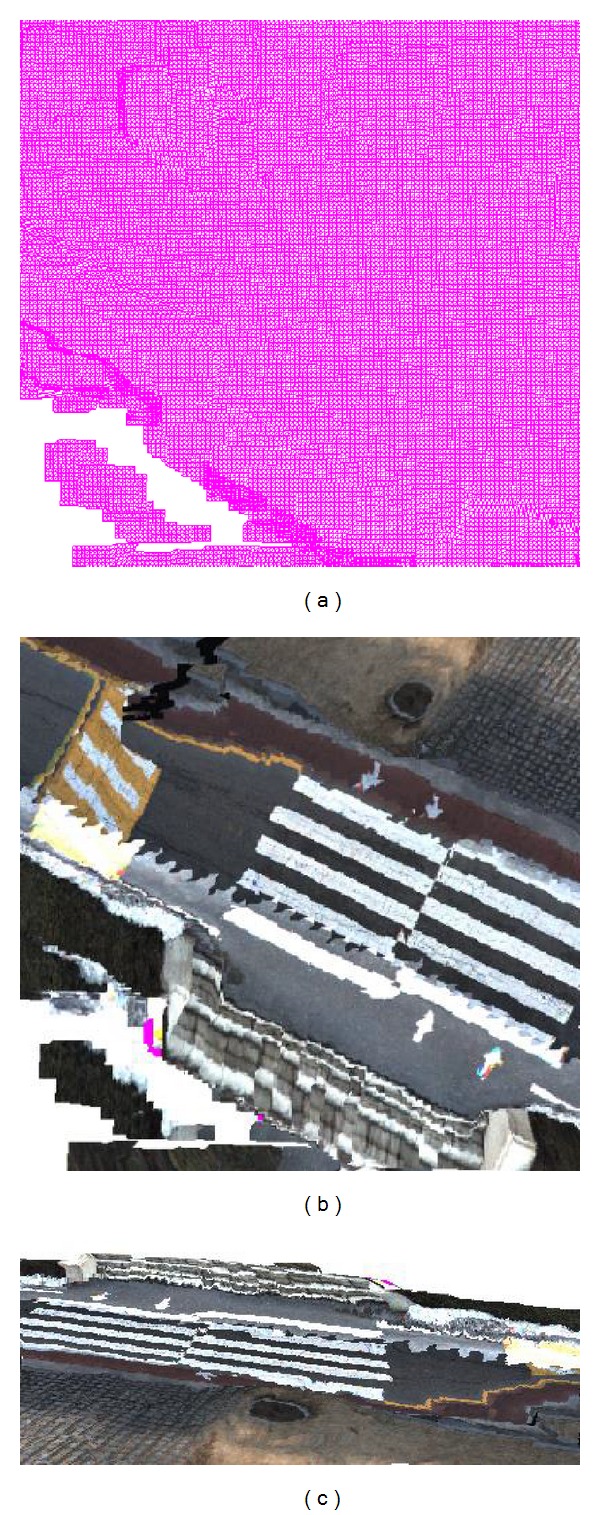
Texture mapping for MDB. (a) A node of the terrain mesh. (b) The node texture of (a). (c) Mapping the node texture onto the node mesh.

We project each triangle in a node mesh, as shown in Figure 5(a), onto the captured 2D images and store the pixel buffers of the mapped triangles in the images. We store these pixel buffers into a node texture, as shown in Figure 5(b), which is combined with the TDB. By mapping the mesh node with its node texture, a texture mesh is generated, as shown in Figure 5(c).

**Figure 6:**
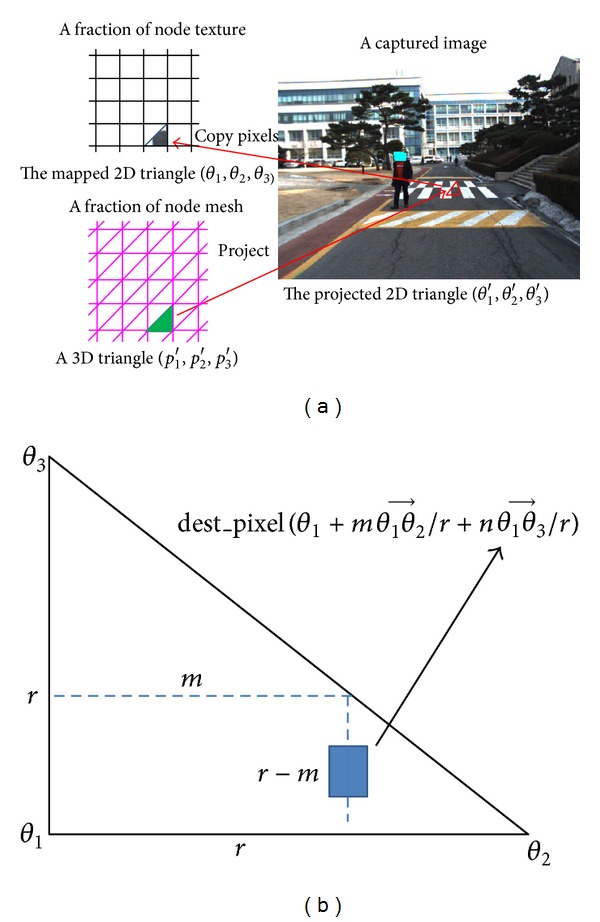
Node texture generation method. (a) Projection from the triangles of a node mesh onto a captured image. (b) Texture triangle duplication.

Figure 6(a) shows the process of node texture generation. For a 3D triangle (*p*_1_′, *p*_2_′, *p*_3_′) in the mesh of that node, we create a 2D triangle (*θ*_1_, *θ*_2_, *θ*_3_) in a node texture, which has a set of triangle pixels. Subsequently, a 2D triangle (*θ*_1_′, *θ*_2_′, *θ*_3_′) in a captured image is projected from the 3D triangle (*p*_1_′, *p*_2_′, *p*_3_′). We then duplicate triangle (*p*_1_′, *p*_2_′, *p*_3_′) from triangle (*θ*_1_′, *θ*_2_′, *θ*_3_′). After all of the triangles in a node mesh are mapped from the captured images, the node texture is updated and combined with the TDB system.

The pseudocode for triangle duplication is as follows:

(3) for(int⁡m=0;m<r;m++) for(int⁡n=0;n<r−m;n++)  dest_pixel(θ1+mθ1θ2→r+nθ1θ3→r)  =sourc_pixel(θ1′+mθ1′θ2′→r+nθ1′θ3′→r),

where *r* is the resolution of the destination triangle and the symbol $\vec{\theta_{1}\theta_{2}}$ stands for a vector from *θ*_1_ to *θ*_2_. As shown in Figure 6(b), each source pixel in a captured image is mapped onto its destination pixel in a node texture.

When the robot navigates a large-scale environment, a large number of mesh nodes are generated and a large number of triangles of these nodes are mapped from the captured video images. To realize real-time TDB generation, we apply GPU programming to implement the mapping process in parallel. The TDB generation process using the GPU is shown in Figure 1. After the current captured images are registered into the GPU memory, we copy the mesh and texture of an updated node and the current captured image to GPU memory. Next, we project each triangle of the node mesh onto the captured images in order to acquire the mapped triangles within the images. Then, we duplicate the mapped triangle to the node texture buffers. Next, we load the updated node texture in the GPU memory to the TDB of the terrain model in the CPU memory. Finally, we render the terrain model by overlaying the MDB with the TDB.

## 4. Experiments and Analysis

In this section, we analyzed the performance of the proposed real-time terrain storage generation and intuitive representation system.

### 4.1. Experimental Setup

**Figure 7:**
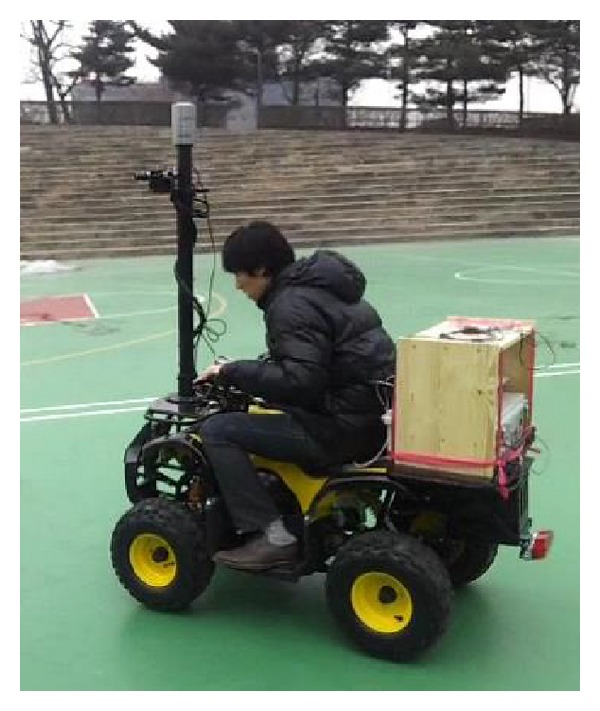
Experimental mobile vehicle.

We carried out the experiments using a mobile robot with integrated sensors, as shown in Figure 7, including a GPS, a gyroscope, three video cameras, and an HDL-32E Velodyne LiDAR sensor. The proposed algorithms were implemented on a computer with a 2.82 GHz Intel Core 2 Quad CPU, a GeForce GTX 275 graphics card, and 4 GB RAM. We drove the robot around a 100 × 100 m^2^ outdoor environment in Dongguk University campus, including vehicles, buildings, and vegetation.

**Figure 8:**
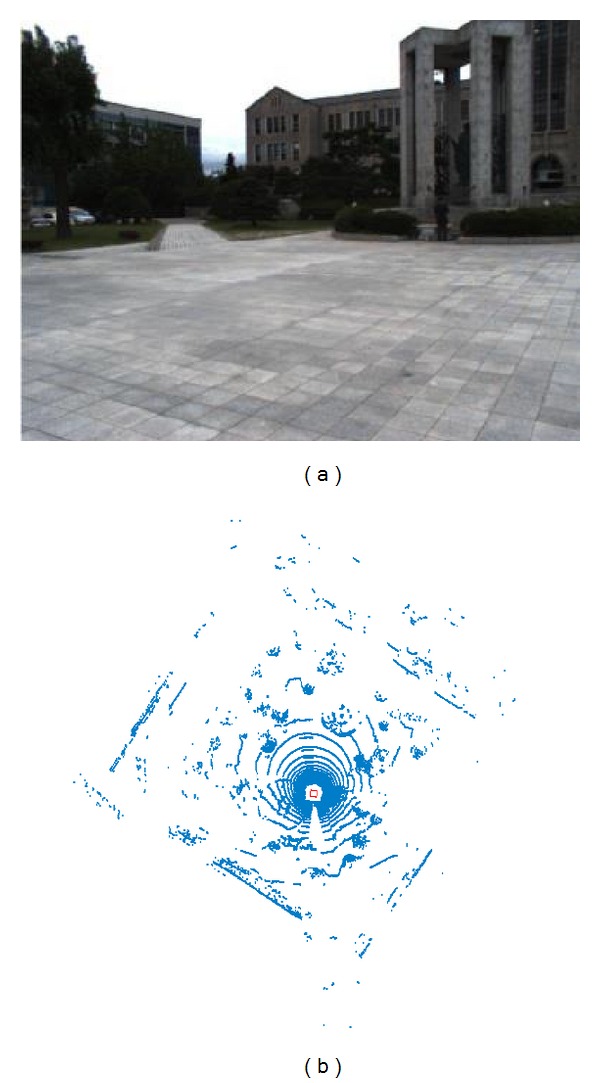
Datasets received from multiple sensors. (a) A captured 2D image. (b) 180 packets of point cloud received from HDL-32E.

We used three GC655 VGA CCD cameras to capture images mounted in front of the robot. We captured RGB-color images with a resolution of 659 × 493 RGB pixels, as shown in Figure 8(a). The HDL-32E provided 32 × 12 points in a packet, for a total packet time of 552.96 *μ*s. It gives approximately 1,808 packets of 3D point clouds per second, which contain 694,292 points. The measurement range is 5–100 m. The valid data range was approximately 70 m from the robot. The field of view is +10.67° to −30.67° in vertical and 360° in horizontal. The angular resolution is 1.33° in vertical. Using the HDL-32E, the received datasets from 180 packets of point clouds are represented in Figure 8(b). The 3D data collection duration is 0.1 s. To realize real-time terrain modeling, the duration of each proposed modeling algorithm needs to be less than 0.1 s for 180 packets.

To integrate measurement sections of LiDAR scans with accurate transformation, we applied a GPS receiver and an inertial measurement unit (IMU) to detect the absolute position and orientation of the LiDAR sensor. In our project, we utilized a GPS-aided, IMU-enhanced MTi-G-700 sensor that offers high-quality orientation and position data. The MTi-G-700 is mounted on the vehicle to report the rotational states, including yaw, pitch, and roll values, and the position, including east, north, and elevation values.

### 4.2. Point Registration Performance Using Voxel-Based Flag Map

**Figure 9:**
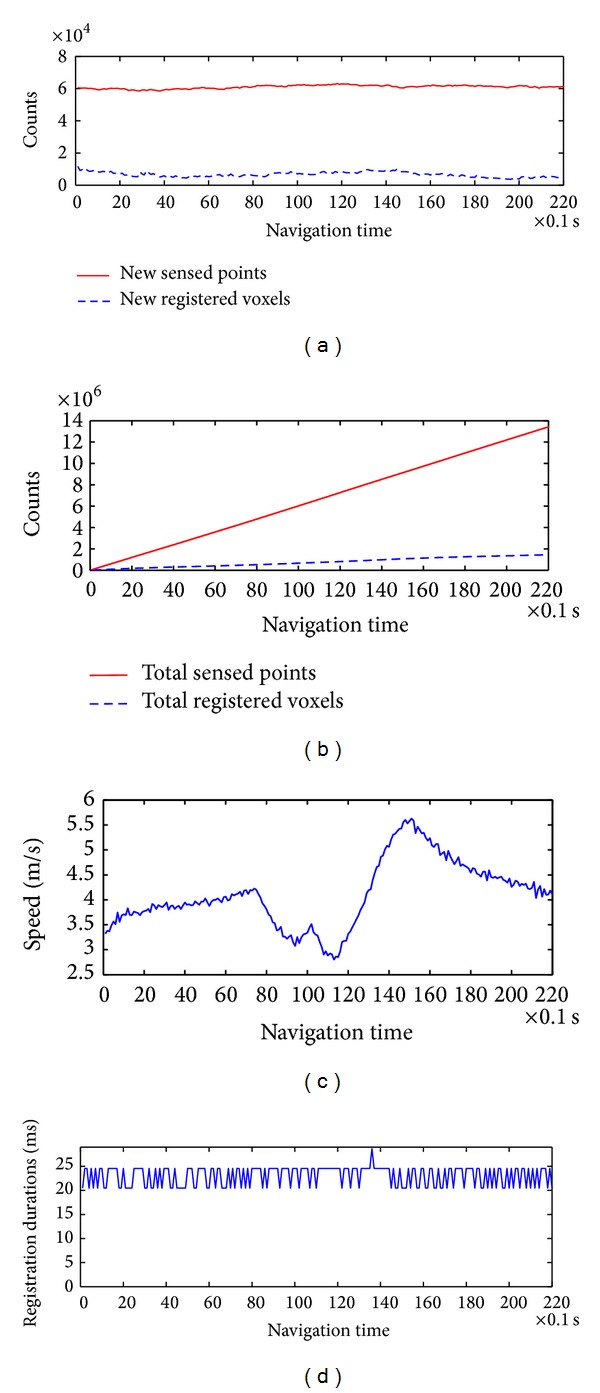
3D point cloud registration using the voxel-based flag map. (a) Counts of new sensed points and registered voxels. (b) Counts of total sensed points and registered voxels. (c) Driving speed. (d) Registration durations of point clouds.

By registering the sensed 3D point clouds into the voxel-based flag map, we remove the redundancy for real-time terrain reconstruction with low memory. In our project, we defined a voxel as a 10 × 10 × 10 cm^3^ cube. We drove the robot around an outdoor area at an average speed of 3.87 m/s. To demonstrate the performance of the flag map, we recorded the counts of the sensed points and the registered voxels, as shown in Figure 9(a).

At the beginning of testing, the flag map was empty. Many points were registered into some voxels that were sensed for the first time. Therefore, the counts of new registered voxels at the beginning are more than those at other navigation times.  Figure 9(b) shows the total sensed points and registered voxels during vehicle navigation. We can see that the flag map removed ~90.06% redundant points from the sensed datasets after the robot navigated for 10 s. Meanwhile, we can see that the faster the vehicle moved, as shown in Figure 9(c), the more voxels in front were sensed and registered.  Figure 9(d) shows the point cloud registration durations during robot navigation. The registration duration for 180 packets was 0.023 s, less than 0.1 s, which satisfied the real-time requirement. When the robot speeded up at the time 12 seconds, there were many new voxels registered in the voxel map. The new registered voxels caused a surge of the terrain database registration duration, as shown in Figure 9(d).

**Figure 10:**
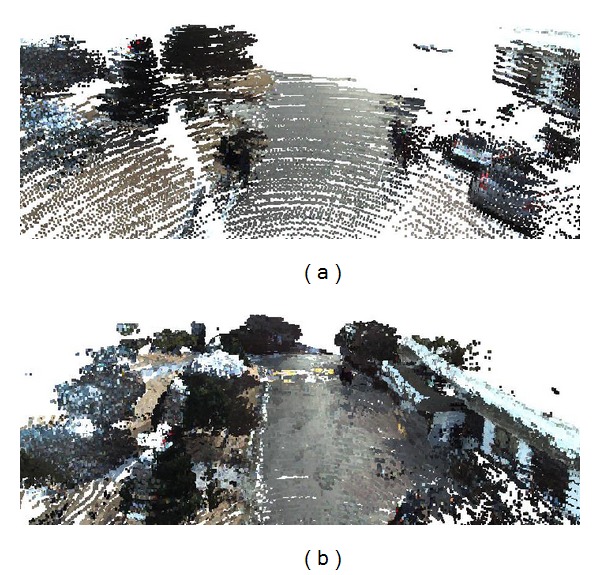
Fractions of generated voxel maps by using the flag map. (a) Registration from 1,808 packets of 3D point clouds. (b) Registration from 18,080 packets of 3D point clouds.

We applied the position of the first registered point to represent a voxel in the terrain model. By using the flag map, we created a voxel map as shown in Figure 10. In Figure 10(a), the sensed validate point count is 600,952 and the number of voxels in the flag map is 91,546. In Figure 10(b), the point count is 6,644,033 and the voxel count is 660,460.

### 4.3. Performances of MDB and TDB Generation Using GPU

**Figure 11:**
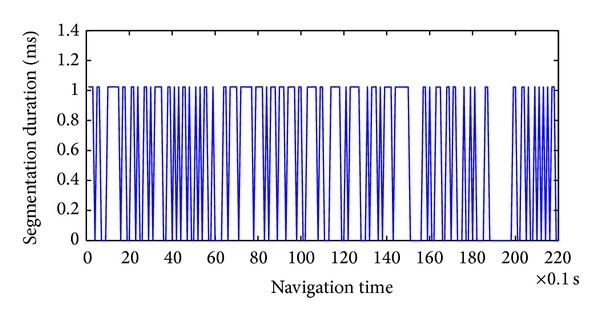
Segmentation duration using height histogram method.

After the voxels were registered in the terrain model, we applied the height histogram method to estimate the height range of the ground surface. Subsequently, we segmented the points into the ground dataset and nonground dataset using the estimated height range as a threshold. In our project, we implemented the ground segmentation procedure once for 180 packets of registered voxels. The ground segmentation duration was 0.5271 ms on average, as shown in Figure 11, which is much faster than 0.1 s and satisfies the real-time requirement.

**Figure 12:**
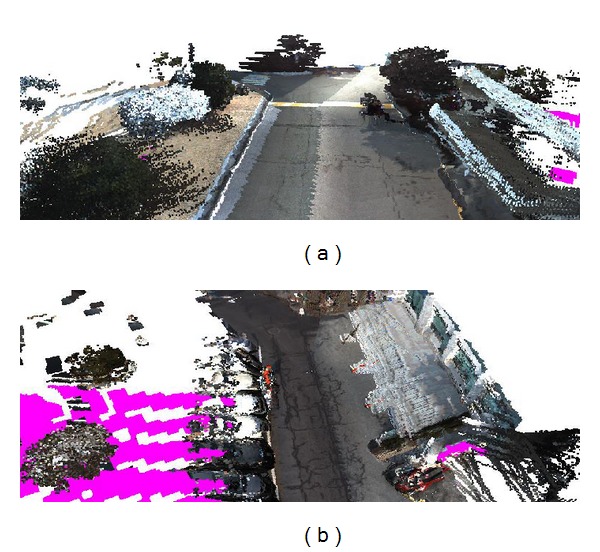
Terrain reconstruction results using ground segmentation method. (a) Nonground voxel map and ground texture mesh representation. (b) Reconstruction result of another environment.

We used a voxel map to represent nonground data and a texture mesh to represent ground data. By projection from the vertices of the terrain model onto the captured images, we computed the color information for the nonground PDB and ground MDB, as shown in Figure 12. After we classified the ground data from the voxels in Figure 10(b), the TDB is generated using the node texture mapping method described in Section 3.4. By overlaying the node textures in the TDB onto the MDB, we represented an intuitive terrain model as shown in Figure 12(a).  Figure 12(b) provides a top view of the terrain model reconstructed at another environment. The regions in the MDB which could not be mapped onto the captured images are represented as the pink regions in Figure 12.

**Figure 13:**
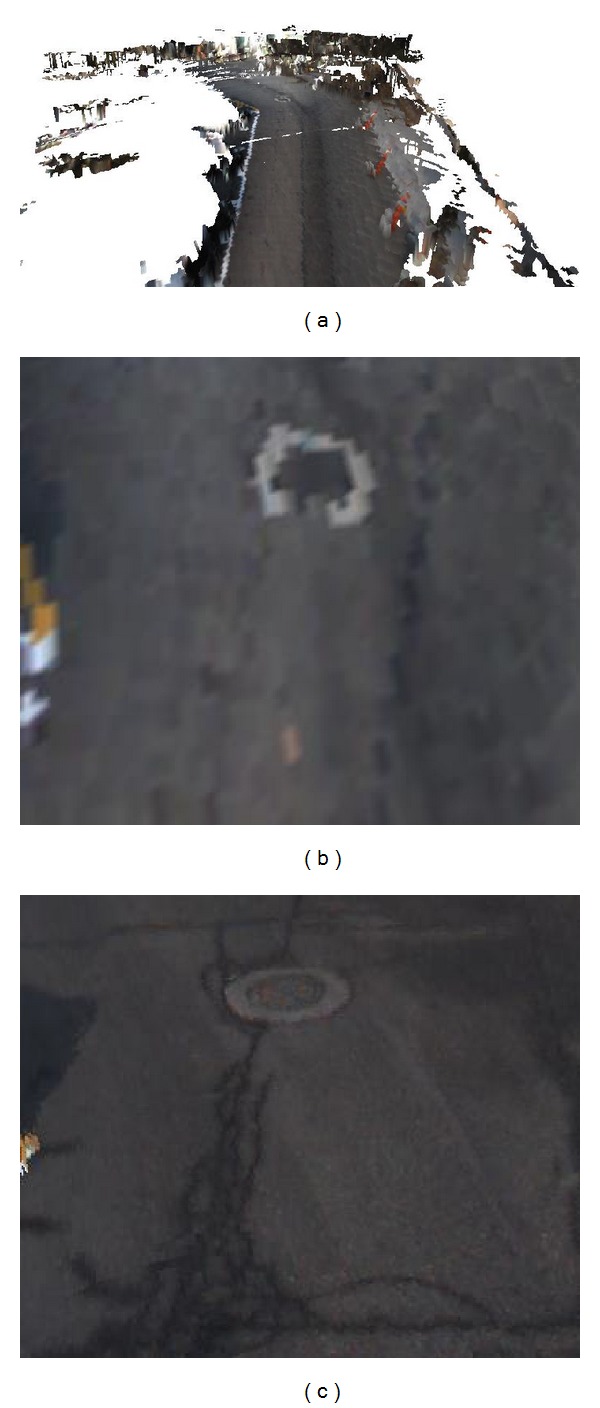
Ground surface representation using a color mesh. (a) A color mesh reconstructed from the segmented ground voxels. (b) A fraction of the color mesh of (a). (c) A fraction of the texture mesh of Figure 12(b).

We captured and registered three images of 659 × 493 pixels every 0.1 s into the memory. If we store the captured images into the memory incrementally, they exceed the computer memory capacity. To solve this problem, Huber et al. [6] applied a color mesh, as shown in Figure 13(a), where a vertex contains RGB color information. As a result, it is not necessary to store the captured images. In our application, a node of the color mesh has 128 × 128 vertices and 128 × 128 colors. Using the color mesh, the projected pixels in the images from the vertices of the mesh were only stored, as shown in Figure 13(b). However, many pixels were lost, causing distortion and a blurry scene. Using the TDB, we defined a texture of 128 × 128 × 4 × 4 XRGB pixels for a node. In this manner, an intuitive ground surface was represented, as shown in Figure 13(c). If the resolution of the node textures increased, the CPU-GPU memory copying speed for updating the node texture buffers became low. To balance the visualization quality and system processing speed, we defined 4 × 4 pixels for a grid in the node textures.

**Figure 14:**
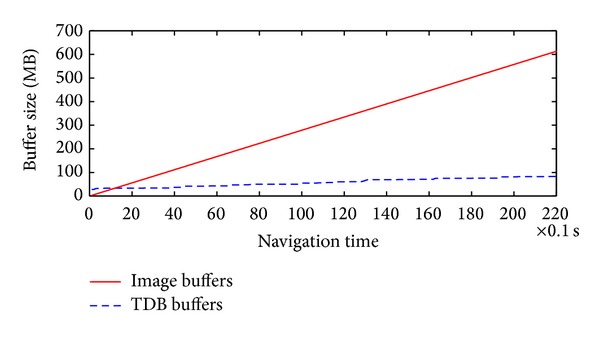
Buffer sizes of the TDB and the captured video images.

We compared the sizes of the TDB and the captured images, as shown in Figure 14. At the beginning of the testing, the robot sensed 28 nodes for the MDB and allocated 28 node textures for the TDB. After 20 s, 600 images were captured and 81 nodes were registered in the MDB. The TDB buffer size of these nodes was 81.0 MB, generated from 557.7 MB of video images. The results demonstrate that the video images captured by the GC655 cameras were registered to the TDB with low memory. In our projects, we rendered 9 nodes of the ground mesh surrounding the robot. The other nodes were stored on a hard disk. Therefore, only 9.31 MB of memory space was used to represent the surrounding ground mesh.

**Figure 15:**
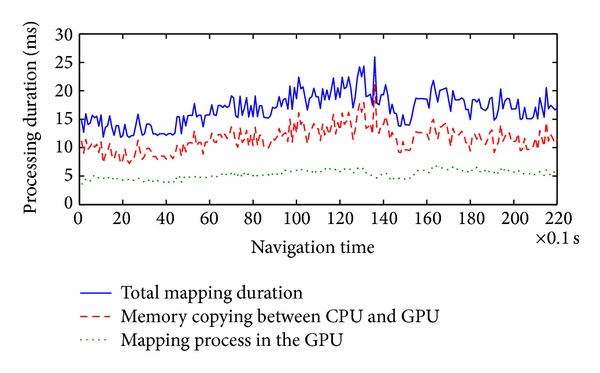
Durations of the mapping process for TDB generation.

To speed up the computation of the TDB generation, we used GPU programming to implement the mapping process in parallel. The duration of the total mapping process was reduced to 17.29 ms on average for every 180 packets, as shown in Figure 15. Because the capability of the applied GTX 275 graphics card is not high, the memory copying process took around 11.83 ms; this was longer than the mapping process in the GPU, which took 5.46 ms on average. TDB generation took much lower than 0.1 s, satisfying the real-time requirement.

**Figure 16:**
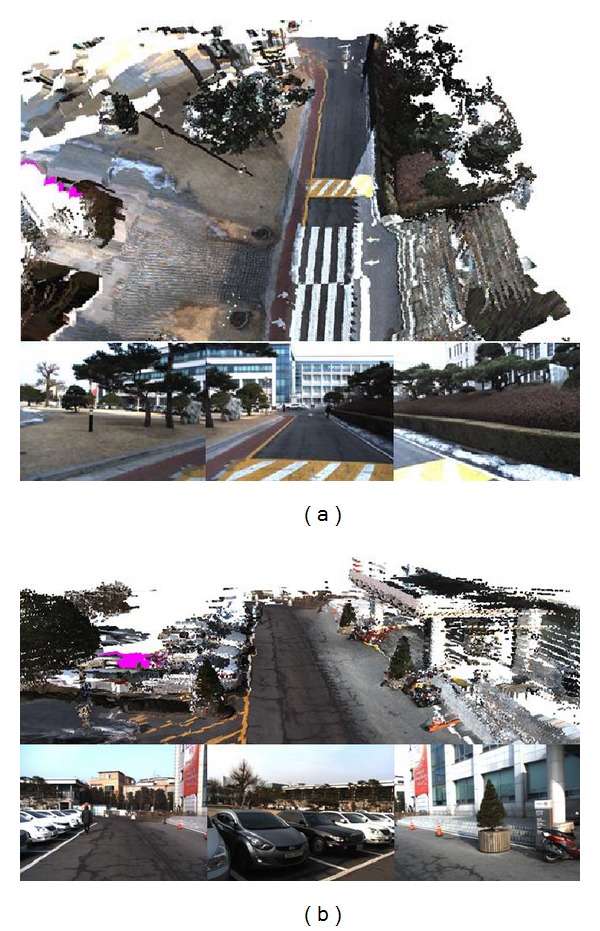
Terrain reconstruction results from the nonground PDB, ground MDB, and TDB. (a) A top view of environment 1. (b) A front view of environment 2.

After updating the nonground PDB, ground MDB, and TDB, we utilized the DirectX SDK to render the terrain model as shown in Figure 16. The three images below the terrain representation results of (a) and (b) were captured by the three cameras.

**Figure 17:**
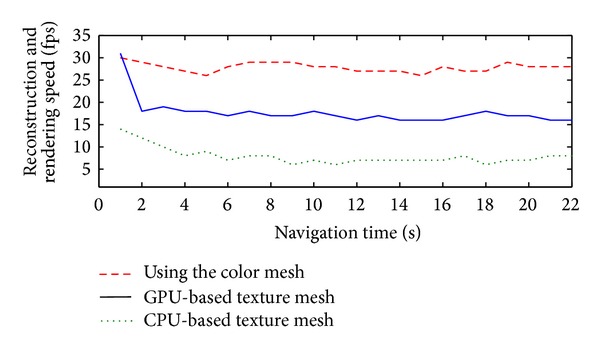
Speed performance comparison of terrain reconstruction and rendering processes using the color mesh, GPU-based, and CPU-based methods.

The terrain reconstruction and visualization speed is shown in Figure 17. We compared the GPU-based texture mesh generation method with the CPU-based method and the color mesh method. Using the CPU, we reconstructed and rendered the terrain model at a speed of 7.12 fps on average. Using the GPU, the speed was improved to 18.85 fps. Although the speed by using the color mesh is around 27.5 fps, the visualization quality is much lower than that by using the texture mesh.

## 5. Conclusions

In this study, we developed a real-time intuitive terrain reconstruction system using a nonground voxel map and a ground texture mesh for automated surveying and mobile mapping services. The mobile robot collects 3D point clouds, 2D images, GPS, and rotation states through multiple sensors. One of our objectives is to register the large-scale point clouds into the terrain model in real time. We proposed a voxel-based flag map to register the 3D points into the terrain model without redundancy. Then, we created a color voxel map to represent nonground PDB and a node-based texture mesh to represent the ground surface. By using the GPU, we realized real-time TDB generation from the large-scale captured images so as to reconstruct the texture mesh with low memory consumption. Finally, we rendered the reconstructed terrain model by overlaying the TDB onto the MDB to provide rapid and intuitive information about the surrounding environment, which provided a GUI between the operators and the mobile robots.

We tested our approach using a mobile robot mounted with integrated sensors in a large-scale environment. Through the flag map, we realized real-time large-scale 3D dataset registration. Using the texture mesh with TDB, we offered an intuitive representation of the reconstructed terrain model, which provides the operator with intuitive visualization support. The time required for terrain reconstruction was faster than that for dataset gathering, which satisfied the real-time requirement.

In our testing result, we found that there were some errors in the GPS and IMU when the robot was shaking. We dropped these packets to remove the errors. Therefore, there were some areas in the voxel map with low density. We need to study the calibration algorithms to adjust these errors in the future.
